# Uncovering noisy social signals: Using optimization methods from experimental physics to study social phenomena

**DOI:** 10.1371/journal.pone.0174182

**Published:** 2017-03-17

**Authors:** Maurits Kaptein, Robin van Emden, Davide Iannuzzi

**Affiliations:** 1 Department of Methodology and Statistics, Tilburg University, Tilburg, The Netherlands; 2 Department of Physics and Astronomy and LaserLab, VU University Amsterdam, The Netherlands; Universidad Nacional de Mar del Plata, ARGENTINA

## Abstract

Due to the ubiquitous presence of treatment heterogeneity, measurement error, and contextual confounders, numerous social phenomena are hard to study. Precise control of treatment variables and possible confounders is often key to the success of studies in the social sciences, yet often proves out of the realm of control of the experimenter. To amend this situation we propose a novel approach coined “lock-in feedback” which is based on a method that is routinely used in high-precision physics experiments to extract small signals out of a noisy environment. Here, we adapt the method to noisy social signals in multiple dimensions and evaluate it by studying an inherently noisy topic: the perception of (subjective) beauty. We show that the lock-in feedback approach allows one to select optimal treatment levels despite the presence of considerable noise. Furthermore, through the introduction of an external contextual shock we demonstrate that we can find relationships between noisy variables that were hitherto unknown. We therefore argue that lock-in methods may provide a valuable addition to the social scientist’s experimental toolbox and we explicitly discuss a number of future applications.

## Introduction

Social science experiments are often affected by large measurement errors [1]. The effects under study are complex [2] and the results of the experiments largely depend on the experimental context [3] or on the particular group of people under study [4]. Due to this complex nature of human behavior, even experiments demonstrating some of the most compelling principles of human decision making have proven difficult to replicate when conditions undergo minor changes or when researchers leave the confines of their laboratories [5, 6]. Hence, it is no surprise that recently there has been an increased interest in the development of experimental methods that are robust to noise or contextual changes. Apart from general guidelines that focus on averting bad research practices [7], these methods range from registering studies and adopting different reporting standards [8–10] to the application of Bayesian statistics [11]. Considerable work has been devoted to optimally choosing possible treatment values to efficiently estimate effects [12–15] (for an extensive overview, we refer the reader to [16]), often focusing on the reduction of variance in estimates obtained given an *a priori* assumed experimental setup and functional relationship between dependent and independent variables [17]. With the functional form of the effect of treatment variables at hand, these methods dictate at which points in treatment space stimuli should be positioned [18]. In recent years, researchers have further turned their attention to *sequential* methods that could determine the optimal design of experiments, the optimal stimuli, or the optimal sample sizes even when the functional form of the effect of a treatment variable is unknown (see for examples [13, 19]). In those cases, treatment assignments are continuously improved as the data are collected [20]. These adaptive designs, and the associated early stopping of experiments [21], currently find application in the health and life sciences [22].

Adding to this vast body of literature, whose systematic review is out of the scope of this paper, in recent work we have demonstrated [23] that, to extract a weak signal out of a noisy floor in a social science experiment, one can also rely on a sequential algorithm similar to the one that drives an electronic piece of equipment often used in high- precision physics experiments—the “lock-in amplifier” [24, 25]. The aim of that work was limited to settling the debate around the efficacy and practical relevance of the so-called “decoy effect” [26, 27]. Given the goal of the experiment, we were able to perform the entire measurement campaign on the basis of a simplified version of the algorithm, which, albeit efficient, was not designed to show the full potential of the method proposed. The algorithm, in fact, was only tested in sequential experiments with one independent variable and one binary dependent variable. In physics and engineering, however, lock-in amplifiers are often utilized in situations where a continuous variable depends on an entire set of independent, continuous variables—a widely used feature in the design of high-precision experiments that often must also be performed within noisy conditions. In this paper, we show that, likewise, the method rudimentarily proposed in [23], which we dubbed as “lock-in feedback” (LiF), can be extended to cover a much broader range of social science experiments than that explored in our first test.

The problem we consider can be described as follows: while, in discrete interactions, data are observed on a number of continuous independent variables that are under the control of the experimenter and on some dependent variable whose value we seek to maximize (or minimize), we need a method to choose, sequentially, the values of our independent variables such that this maximum (or minimum) is both obtained and maintained (the problem can be considered a stochastic optimization problem—see [28] and references therein for an elaborate review). To demonstrate the enabling features of LiF in this context, we selected a topic of study in which heterogeneity and noise abound: we studied the subjective perception of beauty over multiple participants [29, 30]. We confronted participants sequentially with a digital rendering of a face, which can be manipulated in two dimensions (brow-nose-chin ratio and distance between the eyes). We used LiF to find, simultaneously, the values of these two dimensions that—on average—maximize the perception of subjective beauty. We first examined whether LiF finds such an optimum, and subsequently introduce an external shock to see whether LiF is robust. Our results demonstrate that the method can indeed obtain and maintain the maximizing position in the attribute space. Furthermore, we showed that an accurate analysis of the data obtained can reveal interesting and unexpected details on the interplay between the variables of the experiment.

The remainder of this paper is organized as follows: In the next section we describe the mathematics behind LiF for the one-dimensional, continuous, case. In the Methods and Materials section we detail the current empirical study and our specific implementation of LiF in multiple dimensions as used in this trial. The Results section discusses how LiF can distil a signal of subjective beauty from an extremely noisy signal and how it responds to external shocks. In the Discussion we highlight future opportunities for the use of LiF in the social sciences.

### Lock-in feedback circuits

Let us assume that a dependent variable *y* is a continuous function *f* of the independent variable *x*: *y* = *f*(*x*). Let’s further assume that—given that we can manipulate *x*—we can oscillate *x* in time according to:

$$\begin{array}{cc} x(t) & =x_{0}+A\cos\left(\omega t\right) \end{array}$$ (1)

where *ω* is the angular frequency of the oscillation, *x*_0_ its central value, and *A* its amplitude. For relatively small values of *A*, Taylor expanding *f*(*x*) around *x*_0_ to the second order, one obtains:

$$\begin{array}{c} \begin{array}{cc} y(x(t)) & =f\left(x_{0}\right)+\left(x_{0}+A\cos\left(\omega t\right)-x_{0}\right)\left({\left.\frac{\partial f}{\partial x}\right|}_{x=x_{0}}\right)\\ & +\frac{1}{2}{\left(x_{0}+A\cos\left(\omega t\right)-x_{0}\right)}^{2}\left({\left.\frac{\partial^{2}f}{\partial x^{2}}\right|}_{x=x_{0}}\right) \end{array} \end{array}$$ (2)

which can be simplified to:

$$\begin{array}{c} \begin{array}{cc} y(x(t)) & =k+A\cos\left(\omega t\right)\left({\left.\frac{\partial f}{\partial x}\right|}_{x=x_{0}}\right)\\ & +\frac{1}{4}A^{2}\cos\left(2\omega t\right)\left({\left.\frac{\partial^{2}f}{\partial x^{2}}\right|}_{x=x_{0}}\right) \end{array} \end{array}$$ (3)

where *k* = *f*(*x*_0_) + 1/4*A*^2^ (∂^2^*f*/∂*x*^2^|_*x* = *x*_0__). It is thus evident that, for small oscillations, *y* becomes the sum of three terms: a constant term, a term oscillating at angular frequency *ω*, and a term oscillating at angular frequency 2*ω*.

Now consider the case in which *f* is continuous and only has one maximum and no minimum (to keep things relatively simple, we only consider such well-behaved functions in this paper). We are interested in finding the value argmax_*x*_ *y* = *f*(*x*), which we denote with *x*_*max*_, in the presence of noise. Modeling the latter contribution as *ϵ* ∼ *π*(), where *π* is some probability density function and E[ϵ|x]=0, we obtain:

$$\begin{array}{cc} y(t) & =f\left(x\left(t\right)\right)+\epsilon_{t} \end{array}$$ (4)

Following the scheme used in physical lock-in amplifiers [24], we can multiply the observed *y* variable by cos(*ωt*). This is useful since after this multiplication, using Eqs 3 and 4, one obtains:

$$\begin{array}{c} \begin{array}{cc} y_{\omega }\left(t\right) & =\cos\left(\omega t\right)[k+A\cos\left(\omega t\right)\left({\left.\frac{\partial f}{\partial x}\right|}_{x=x_{0}}\right)\\ & +\frac{1}{4}A^{2}\cos\left(2\omega t\right)\left({\left.\frac{\partial^{2}f}{\partial x^{2}}\right|}_{x=x_{0}}\right)+\epsilon ]. \end{array} \end{array}$$ (5)

This can be written more compactly as:

$$\begin{array}{c} \begin{array}{cc} y_{\omega } & =\frac{A}{2}\left({\left.\frac{\partial f}{\partial x}\right|}_{x=x_{0}}\right)\\ & +k_{\omega }\cos\left(\omega t\right)+k_{2\omega }\cos\left(2\omega t\right)\\ & +k_{3\omega }\cos\left(3\omega t\right)+\epsilon \cos\left(\omega t\right) \end{array} \end{array}$$ (6)

where

$$\begin{array}{cc} k_{\omega } & =k+A^{2}/8\left({\left.\partial^{2}f/\partial x^{2}\right|}_{x=x_{0}}\right) \end{array}$$ (7)

$$\begin{array}{cc} k_{2\omega } & =A/2\left({\left.\partial^{2}f/\partial x^{2}\right|}_{x=x_{0}}\right) \end{array}$$ (8)

$$\begin{array}{cc} k_{3\omega } & =A^{2}/8\left({\left.\partial^{2}f/\partial x^{2}\right|}_{x=x_{0}}\right). \end{array}$$ (9)

Next, by integrating *y*_*ω*_ over a time $T=\frac{2\pi N}{\omega }$, where *N* is a positive integer and *T* denotes the time needed to integrate *N* full oscillations, one obtains:

$$\begin{array}{c} y_{\omega }^{*}=\frac{TA}{2}\left({\left.\frac{\partial f}{\partial x}\right|}_{x=x_{0}}\right)+\int_{0}^{T}\epsilon \cos\left(\omega t\right)\;dt \end{array}$$ (10)

Depending on the noise level, we are able to tailor the integration time, *T*, in such a way that we can reduce the second addendum of the right hand of Eq 10 to negligible levels, effectively averaging out the noise in the measurements. Under these circumstances, $y_{\omega }^{*}$ provides a direct measure of the value of the first derivative of *f* at *x* = *x*_0_.

**Fig 1:**
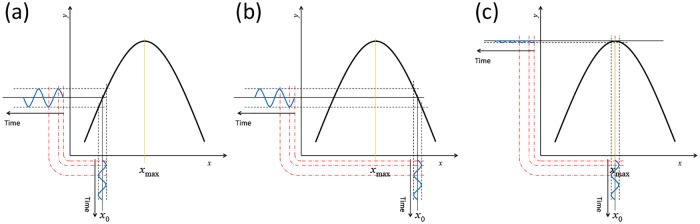
Graphical illustration of LiF. LiF moves and maintains an independent controllable variable *x* onto the value *x*_*max*_ for which a dependent variable *y* is maximized. The value of *x* is oscillated sinusoidally around a central value *x*_0_. (a): If *x*_0_ < *x*_*max*_, *y* oscillates at an equal frequency as *x*, in phase (that is, a maximum value of *x* corresponds to a maximum value of *y*). (b): If *x*_0_ > *x*_*max*_, *y* oscillates again at the same frequency as *x*, but with an opposite phase (that is, a maximum value of *x* corresponds to a minimum value of *y*). (c): If *x*_0_ = *x*_*max*_, *y* ceases to oscillate at the frequency of *x*, but will now start to oscillate at a doubled frequency. LiF can detect the amplitude and the phase of the oscillation at a reference frequency, and is therefore able to indicate whether *x* is smaller, larger, or equal to *x*_0_.

This latter fact provides a logical sequential update strategy for *x*_0_: if $y_{\omega }^{*}<0$, then *x*_0_ is larger than the value of *x* that maximizes *f*; likewise, if $y_{\omega }^{*}>0$, *x*_0_ is smaller than the value of *x* that maximizes *f*. Thus, based on the oscillation observed in *y*_*ω*_ we are now able to move *x*_0_ closer to *x* = argmax_*x*_ *f*(*x*) using an update rule $x_{0}\;≔\;x_{0}+\gamma y_{\omega }^{*}$ where *γ* quantifies the learn rate of the procedure. Hence, we can setup a feedback loop that allows us to keep *x*_0_ close to *x*_*max*_. Note that due to the continuous oscillations around *x*_0_ LiF effectively keeps “checking” whether the derivative of *f*() changes; this allows one to follow possible changes in *x*_*max*_ over time. To summarize, Fig 1 introduces LiF graphically: by systematically oscillating *x* we gain direct information regarding the derivative of *y* even in situations with large noise. We can subsequently use this information to optimally position *x*.

## Materials and methods

In our evaluation of the utility of LiF for the social sciences, which was conducted online, we asked *N* = 7402 participants to express their opinion on the physical attractiveness of an avatar’s face (the dependent variable *y*). All faces were identical, except for the brow-nose-chin ratio (first independent variable *x*_1_) and the eye-to-eye distance (second independent variable *x*_2_). Our goal was to use LiF to sequentially and simultaneously determine the values of *x*_1_ and *x*_2_ that maximize *y*.

### Participants

*N* = 7414 participants were recruited on Amazon Mechanical Turk—a web-based tool that has been recognized as a trustworthy platform for social science experiments [31, 32]. We used its built-in system of qualifications to ensure that only people with an approval rate of at least 90% and at least 100+ completed prior tasks on that platform were allowed to participate. After providing consent, participants could log in, perform the task as described above, fill in a non-mandatory set of demographic questions, and receive a monetary compensation (.40 USD) for their participation in the study. The study was part of a larger online survey consisting of 8 unrelated decision tasks of which the current task was the last, and the other seven are not reported here.

Of our *N* = 7414 participants, *N* = 7402 completed the facial attractiveness task. Of these, *N* = 21 did not fill out the demographics questions. Of the remaining 7381 participants, the largest group (42.4%) was between 25 and 34 years old. All participants were older than 18, and 1.8% of our participants was older than 65. Furthermore, 48.0% of the participants was female. The vast majority of our participants resided in the United States (98.4%), and 89.1% received an education past the high school level.

### Data availability

All the data generated in this study, including the demographics, are available in the replication package which can be found at http://dx.doi.org/10.7910/DVN/Q0LJVI [33].

### Materials

As noted above, the experiment was conducted online through Mechanical Turk. Here we describe in detail the stimuli used (e.g., the rendered face), and the obtained measures.

#### Stimulus

**Fig 2:**
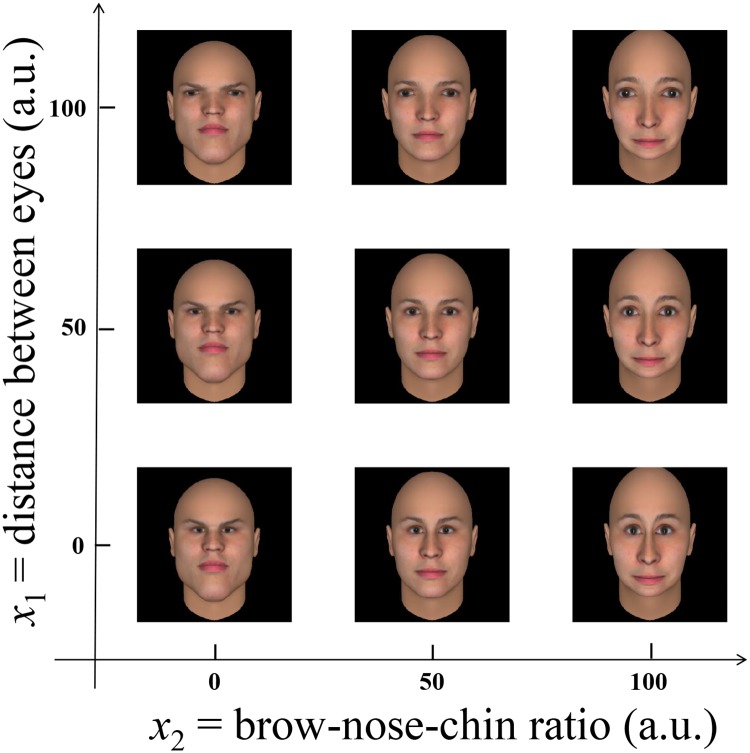
Schematic representation of the stimulus used to examine the performance of LiF. Each of the faces is obtained by either increasing or decreasing the distance between the eyes (denoted *x*_1_ in Methods section) or the elongation of the face (*x*_2_).

To quantify the attribute space, we generated a grid of 100 × 100 faces corresponding to 100 different values of *x*_1_ and *x*_2_. Fig 2 illustrates the resulting metrics. All faces were obtained by means of FaceGen Modeler [34]. We used the “default” face as shipped with the software—which is itself an average of a large set of facial models that is known to be attractive [29]—as a starting point (the middle face in Fig 2). Next, we adjusted the brow-nose-chin ratio and the distance between the eyes to create the outer images (*x*_1_ = 1 or *x*_1_ = 100 and *x*_2_ = 1 or *x*_2_ = 100), and subsequently used FantaMorph [35] to create intermediary faces. The resulting 10000 images, and a javascript library to render the faces as a function of the attributes, can be found in the replication package of this study.

**Fig 3:**
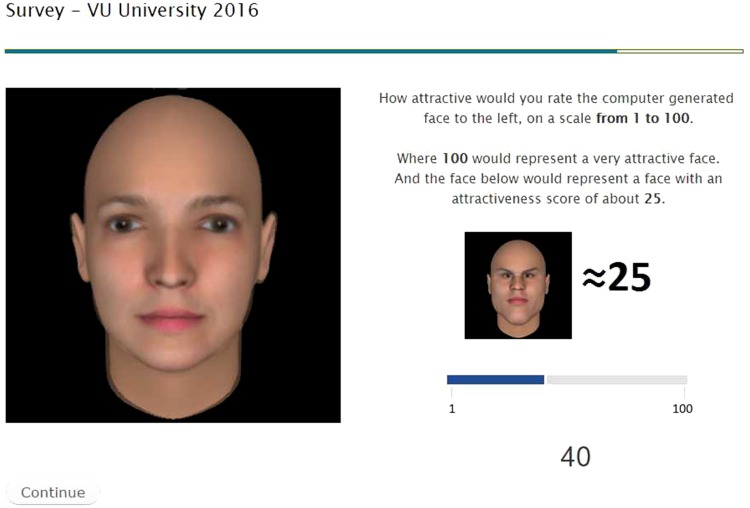
Example of the web page shown to our participants. Except for the left avatar, the design and setup of the web page remained the same throughout the experiment. For the avatar, the brow-nose-chin ratio and eye-to-eye distance were adjusted according to the LiF output. Participants could express their opinion via the slider on the bottom.

Fig 3 shows the primary screen of our experiment. On the left side of the screen, participants saw the face they were asked to evaluate, whose attributes were sequentially adjusted according to the LiF algorithm, as explained later in the text. LiF was implemented using a software package for sequential experiments called StreamingBandit [36], which is publicly available at https://github.com/MKaptein/streamingbandit.

#### Measurements

The main measurement in this study was the rating of subjective beauty of the rendered face (*y*). This subjective evaluation was measured using a slider (see Fig 3, bottom) that ran from 1 (not attractive) to 100 (very attractive). To anchor the scores and explain the scale usage, we presented an example face with the notice that the attractiveness of this face—which was the same for every participant—was approximately 25. Upon arrival on the page the slider was positioned at a value of 40 and participants could move the slider around before confirming their answer by clicking “continue”.

On clicking the “continue” button, participants were asked to complete the study by filling out their gender, age category (18−24, 25−34, 35−44, 45−54, 55−64, 65+), country of residence, and highest completed education. Note that filling out these demographic questions was not obligatory.

### LiF implementation

Given the construction procedure of the face, it is legitimate to assume that there exist a value of *x*_1_ (brow-nose-chin ratio) and a value of *x*_2_ (distance between the eyes) for which the appearance of the face maximizes the average attractiveness score $\bar{y}$. We will indicate those two maximizing values with *x*_1*M*_ and *x*_2*M*_. Our goal is to find those two *a priori* unknown values using LiF. Here we describe how we extended the general LiF method to find an optimum in two dimensions. For the sake of simplicity, we will assume that, close to *x*_1*M*_ and *x*_2*M*_:

$$\begin{array}{c} y\left(x_{1},x_{2}\right)=A_{1}\;{\left(x_{1}-x_{1M}\right)}^{2}+y_{10}+A_{2}\;{\left(x_{2}-x_{2M}\right)}^{2}+y_{20} \end{array}$$ (11)

where *x*_1*M*_, *x*_2*M*_, *A*_1_, *A*_2_, *y*_10_, and *y*_20_ are unknown constants. Let us suppose that the values of *x*_1_ and *x*_2_ as seen by the *i*^*th*^ participant are selected according to:

$$\begin{array}{c} x_{1,i}={\tilde{x}}_{1,i}+\delta_{1}\cos\left(\omega_{1}i\right) \end{array}$$ (12)

$$\begin{array}{c} x_{2,i}={\tilde{x}}_{2,i}+\delta_{2}\cos\left(\omega_{2}i\right) \end{array}$$ (13)

where *i* ranges from 1 to the total number of participants *N*; ${\tilde{x}}_{1,1}$, ${\tilde{x}}_{2,1}$, *ω*_1_, *ω*_2_, *δ*_1_, and *δ*_2_ are six suitably chosen constants set at the start of the experiment; and ${\tilde{x}}_{1,i}$ and ${\tilde{x}}_{2,i}$ have to be sequentially adjusted to find the value of *x*_1*M*_ and *x*_2*M*_. Note that, in this way, we are building the premises to make LiF run on the sequential number of the participants (*i*) in lieu of real-time. In other words, the concept of oscillation period is not to be intended as the interval of time needed to complete the sinusoidal cycle but as the number of people who have to respond to the stimulus to complete the sinusoidal cycle, regardless the time it will take for those people to take that action. Plugging Eqs 12 and 13 into Eq 11, one can conclude that the expected response of the *i*^*th*^ participant is given by:

$$\begin{array}{c} \begin{array}{cc} y_{i}^{expected}= & A_{1}\;{\left({\tilde{x}}_{1,i}+\delta_{1}\cos\left(\omega_{1}i\right)-x_{1M}\right)}^{2}+y_{10}+\\ & A_{2}\;{\left({\tilde{x}}_{2,i}+\delta_{2}\cos\left(\omega_{2}i\right)-x_{2M}\right)}^{2}+y_{20}+\xi_{i} \end{array} \end{array}$$ (14)

where we have added the term *γ*_*i*_ to include the noise generated by the personal preference of the *i*^*th*^ participant. Eq 14 yields:

$$\begin{array}{c} \begin{array}{cc} y_{i}^{expected}= & 2A_{1}\;\left({\tilde{x}}_{1,i}-x_{1M}\right)\delta_{1}\cos\left(\omega_{1}i\right)+\\ & 2A_{2}\;\left({\tilde{x}}_{2,i}-x_{2M}\right)\delta_{2}\cos\left(\omega_{2}i\right)+\\ & A_{1}\;{\left({\tilde{x}}_{1,i}-x_{1M}\right)}^{2}+A_{2}{\left({\tilde{x}}_{2,i}-x_{2M}\right)}^{2}+\\ & \frac{A_{1}\delta_{1}^{2}\cos\left(2\omega_{1}i\right)}{2}+\frac{A_{2}\delta_{2}^{2}\cos\left(2\omega_{2}i\right)}{2}+\\ & \frac{A_{1}\delta_{1}^{2}}{2}+\frac{A_{2}\delta_{2}^{2}}{2}+y_{10}+y_{20}+\xi_{i} \end{array} \end{array}$$ (15)

Note that the amplitude of the oscillations at *ω*_1_ is proportional to how far the attribute *x*_1_ is from the ideal value. Similarly, the amplitude of the oscillations at *ω*_2_ is proportional to how far the attribute *x*_2_ is from the ideal value. One can thus use a LiF to isolate these contributions from the others and drive a feedback circuit to sequentially bring ${\tilde{x}}_{1}$ and ${\tilde{x}}_{2}$ closer and closer to *x*_1*M*_ and *x*_2*M*_, respectively.

Following this approach, at the start of the experiment we first collect the value of *y* for the first *n*_1_ participants, where *n*_1_ is a constant number set *a priori*, with *n*_1_ << *N*. During this first phase, ${\tilde{x}}_{1,i}$ is kept constant: ${\tilde{x}}_{1,1...n_{1}}={\tilde{x}}_{1,1}$. For each value of *i* from 1 to *n*_1_, we multiply the experimental value of *y* times cos(*ω*_1_ *i*), and sum the resulting products from *i* = 1 to *i* = *n*_1_:

$$\begin{array}{c} y_{lock1,n_{1}}^{exper}=\sum_{i=1}^{n_{1}}y_{i}^{exper}\cos\left(\omega_{1}i\right) \end{array}$$ (16)

Following the working principle of LiF, we then use the result of Eq 16 to set the value of ${\tilde{x}}_{n_{1}+1}$:

$$\begin{array}{c} {\tilde{x}}_{1,n_{1}+1}=\frac{\sum_{i=1}^{n_{1}}{\tilde{x}}_{1,i}}{n_{1}}-\gamma_{1}y_{lock1,n_{1}}^{exper} \end{array}$$ (17)

where *γ*_1_ is a constant that we fixed *a priori*. Then, after the (*n*_1_ + 1)^*th*^ participant has answered, we calculate the summation of Eqs 16 and 17 for *i* that goes from 2 to *n*_1_ + 1, and apply the same procedure to determine the values of ${\tilde{x}}_{1,n_{1}+2}$. Iterating the procedure further via the generic equations:

$$\begin{array}{c} y_{lock1,j}^{exper}=\sum_{i=j-n_{1}+1}^{j}y_{i}^{exper}\cos\left(\omega_{1}i\right) \end{array}$$ (18)

and

$$\begin{array}{c} {\tilde{x}}_{1,j+1}=\frac{\sum_{i=j-n_{1}+1}^{j}{\tilde{x}}_{1,i}}{n_{1}}-\gamma_{1}y_{lock1,j}^{exper} \end{array}$$ (19)

one should observe that the value of ${\tilde{x}}_{1,i}$ eventually reaches *x*_1*M*_. Applying, in parallel, a similar algorithm to the variable *x*_2_, one can simultaneous bring ${\tilde{x}}_{2,i}$ to *x*_2*M*_.

To understand why the feedback loop described above should converge to the optimal values, one can calculate the expected signal that the lock-in algorithm should give if the experimental values of *y* followed exactly the expected trend ($y_{i}^{exper}=y_{i}^{expected}$). Plugging Eq 15 into Eq 18, one obtains:

$$\begin{array}{c} y_{lock1,j}^{expected}=A_{1}\delta_{1}\sum_{i=j-n_{1}+1}^{n_{1}}\left({\tilde{x}}_{1,i}-x_{1M}\right)+o.t. \end{array}$$ (20)

where *o*.*t*. indicates terms that, for a sufficiently large value of *n*_1_, become negligible. Inverting Eq 20, one can indeed verify that:

$$\begin{array}{c} x_{1M}\approx \frac{\sum_{i=j-n_{1}+1}^{n_{1}}{\tilde{x}}_{1,i}}{n_{1}}-\frac{y_{lock1,j}^{expected}}{A_{1}\delta_{1}n_{1}}. \end{array}$$ (21)

For a suitable choice of *γ*_1_, *γ*_2_, *δ*_1_, and *δ*_2_, the algorithm presented should thus be able to complete the task. Table 1 presents our choices for tuning parameters used in our experiment.

**Table 1:** Values of the tuning parameters used for the LiF algorithm in this study.

Lock-in 1	*ω*_1_ = 2.63; *n*_1_ = 150; *δ*_1_ = 8; *γ*_1_ = 0.0006
Lock-in 2	*ω*_2_ = 2.51; *n*_2_ = 150; *δ*_2_ = 8; *γ*_2_ = 0.0006

### Ethics statement

Our experimental procedure was approved by the Research Ethics Review Board of the Faculty of Economics and Business Administration of the VU Universiteit Amsterdam.

## Results

**Fig 4:**
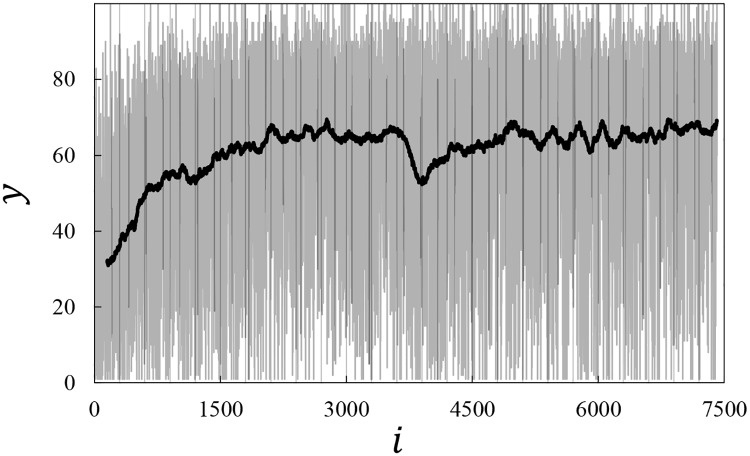
Raw answers on the rating scale. Grey line: Evolution of the observed attractiveness *y* as a function of the participant number *i*. Black line: Same data after taking a running average over 150 participants.

Our experiment had two objectives. First, we intended to test whether LiF would indeed converge towards an optimal value of two treatments simultaneously in the face of considerable noise. Second, we wanted to examine whether LiF would be able to withstand external shocks. Fig 4 displays the raw answers on the rating scale as provided by our *N* = 7402 participants in sequence. The gray line shows the raw scores and illustrates lucidly the extremely noisy setting: raw ratings range from 0 to 100 at almost any configuration of the actual face. The solid black line presents a moving average rating over a sample of 150 participants; this line clearly describes an upwards trend—indicating increasing average attractiveness—over the first 2000 data points after which the (average) ratings seem to stabilize. The “dip” in mean ratings around *i* = 3750 is caused by our external shock, as described later in the text.

**Fig 5:**
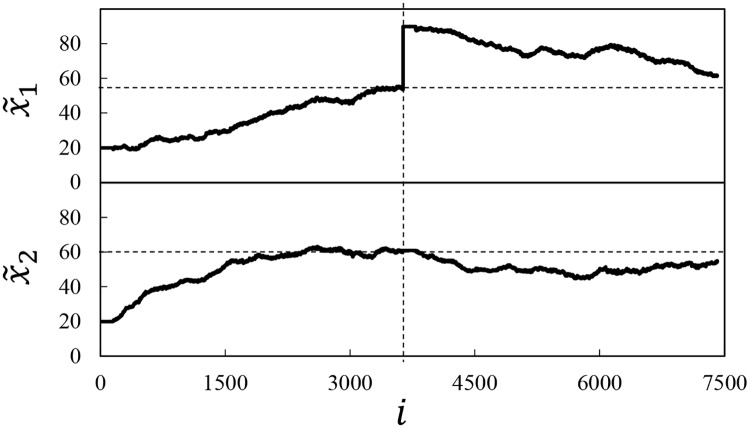
Evolution of ${\tilde{x}}_{1}$ and ${\tilde{x}}_{2}$ as a function of the participant number *i*. The vertical dashed lines indicate the instant in which we forced ${\tilde{x}}_{1}=90$ (*i* = 3637). The two horizontal lines indicate the values of ${\tilde{x}}_{1}$ and ${\tilde{x}}_{2}$ that optimize the avatar’s appearance as obtained from the first phase of the experiment. The avatars below the graph show the starting and arriving points of the two phases of the experiment.

To inspect the performance of LiF for choosing the treatment values that maximize the (average) perceived subjective attractiveness of the rendered face, in Fig 5 we report the values of ${\tilde{x}}_{1,i}$ and ${\tilde{x}}_{2,i}$ and their progression as participants sequentially rate the attractiveness of the face. In the first phase of the experiment, we set ${\tilde{x}}_{1,1}=20$ and ${\tilde{x}}_{2,1}=20$, and let LiF run until *i* = 3636. By this time LiF seems to have converged quite convincingly around values of ${\tilde{x}}_{1}\approx 55$ and ${\tilde{x}}_{2}\approx 60$—in agreement with the literature on subjective beauty [37]. These results demonstrate the ability of LiF to find optimal treatments values in this extremely noise scenario (first goal of our paper).

Our second objective was examined by introducing a shock at *i* = 3636; at this point in time we set ${\tilde{x}}_{1,3637}=90$, and observed the lock-in feedback recovering from this perturbation until *i* = *N* = 7402. Fig 5 clearly shows how LiF “recovers” quickly from the perturbation, and finds the optimal value of the treatment; hence, LiF is able to both position treatments sequentially and respond aptly to (contextual) shocks.

Finally, it is interesting to note that as soon as we set ${\tilde{x}}_{1}=90$, the variable ${\tilde{x}}_{2}$, which was already optimized in the first phase of the experiment, starts to decrease before moving back towards the optimal value. We believe that this behavior is due to the fact that the true function that connects *y* with *x*_1_ and *x*_2_, which we simplified as the sum of two independent parabolas in Eq 11, also involves cross terms that mix the two variables. Hence, the optimal value of *x*_2_ actually depends on the current value of *x*_1_. This finding uncovers a—to our best knowledge—not previously reported dependence between the brow-chin-nose ratio and the eye-distance in their joint effect on the attractiveness of a face. Apparently, for a large distance between the eyes, faces with slightly smaller brow-nose-chin ratio are preferred. Thus LiF, even while treating both attributes independently, allowed us to demonstrate a dependency between the two attributes manipulated in this study.

## Conclusions

We have shown how the algorithm of lock-in feedback amplifiers, which is routinely used in high-precision physics experiments [38], can be applied to social science experiments. In this setting the algorithm allows experimenters to optimally choose treatment values in a multidimensional treatment space even in the face of large noise. Furthermore, we have demonstrated that this approach can quickly recover from external perturbations—an important feature that increases its potential for social science experiments in which contextual changes are likely to introduce such external perturbations. In the current study we track the (group)-average subjective evaluation of beauty; we assume that this is relatively constant within the study given shared timing and context. LiF would theoretically be able to measure fluctuations in the subjective experience within individuals if their opinions were measured sequentially over time; an approach not further explored here. Finally, we have demonstrated that the method can unveil non-trivial, unexpected correlations between the variables involved in a social experiment.

LiF potentially provides a simple-to-implement, effective, and robust method to any situation in which either the value of (a set of) dependent variable(s), or of a possible confounding variable, needs to be set such that the effect under study (or some function thereof) is maximized (or minimized). Examples include, but are not limited to, determining the value of continuous treatments in economic decision experiments (offered prices, product features, etc. [39]), determining optimal dosages of medical treatments, determining optimal values of health promotion feedback (see [40]), or choosing the speed at which stimuli are displayed in reaction tasks such that effects are magnified (such as [41]). Note that LiF can be used not only to position treatments during experiments but can also be of use in practical applications [23].

Interestingly, lock-in feedback might even shed light on the relationship between different variables. In the current paper we uncovered a relationship between the brow-chin-nose ratio and the eye-distance that has not been reported before. Other fields of applications may include the design of optimal strategies in game theory and the analysis of correlations in network. Note that studying this relationship by means of a conventional experiment would have been challenging; one would have to a) discretize the two independent variables to create a grid of possible combinations of values, and b) obtain a large number of observations within each cell to average out the large noise. This would quickly lead to a necessity of an extremely large subject pool, or, conversely, to low power. Since LiF was already operating in a sensitive region of parameter space, the method allowed for finding a novel relationship quite effectively.

We believe our work demonstrates the feasibility of LiF as a versatile sequential treatment selection method in the social sciences. Potentially, the use of LiF will aid replicability of social science findings, and contribute to a greater external validity of findings by allowing precise choice of treatment in multiple contexts.
